# Platelet Extracellular Vesicles as Natural Delivery Vehicles for Mitochondrial Dysfunction Therapy?

**DOI:** 10.1021/acsbiomaterials.5c00473

**Published:** 2025-04-25

**Authors:** Hsien Chang Yeh, Kirti Gupta, Ya-Hsuan Lu, Abinaya Srinivasan, Liling Delila, Nguyen Tran Hai Yen, Ariunjargal Nyam-Erdene, Thierry Burnouf

**Affiliations:** † School of Medicine, College of Medicine, Taipei Medical University, Xin-Yi Campus, Taipei City 110, Taiwan; ‡ International Graduate Program in Medicine, College of Medicine, Taipei Medical University, Xin-Yi Campus, Taipei 110, Taiwan; § School of Biomedical Engineering, Taipei Medical University, Shuang-Ho Campus, New Taipei City 110, Taiwan; ∥ International PhD Program in Biomedical Engineering, College of Biomedical Engineering, Taipei Medical University, Shuang-Ho Campus, New Taipei City 110, Taiwan; ⊥ Graduate Institute of Biomedical Materials and Tissue Engineering, College of Biomedical Engineering, Taipei Medical University, Shuang-Ho Campus, New Taipei City 110, Taiwan; # International PhD Program in Cell Therapy and Regeneration Medicine, College of Medicine, Taipei Medical University, Taipei 110, Taiwan

**Keywords:** extracellular vesicles, platelet, exosomes, microvesicles, oxidative stress

## Abstract

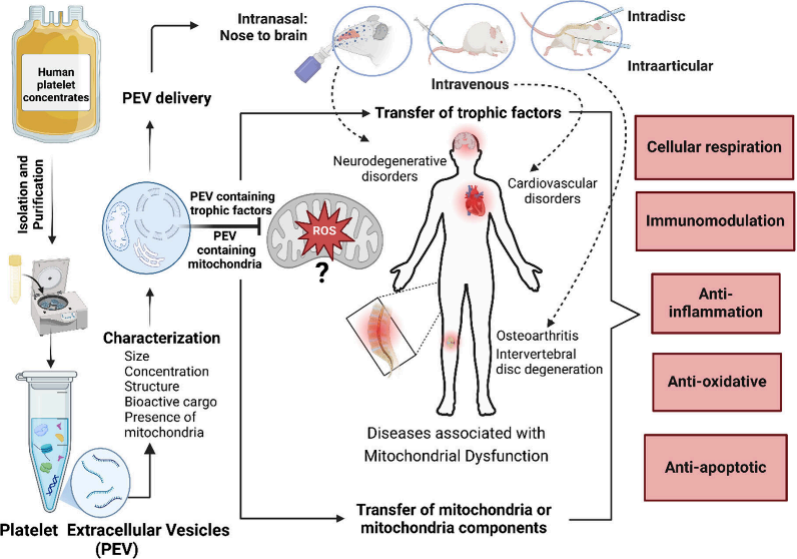

Mitochondria are vital for energy production, metabolic regulation, and cellular signaling. Their dysfunction is strongly implicated in neurological, cardiovascular, and muscular degenerative diseases, where energy deficits and oxidative stress accelerate disease progression. Platelet extracellular vesicles (PEVs), once called “platelet dust”, have emerged as promising agents for mitigating mitochondrial dysfunction. Like other extracellular vesicles (EVs), PEVs carry diverse molecular cargo and surface markers implicated in disease processes and therapeutic efficacy. Notably, they may possibly contain intact or partially functional mitochondrial components, making them tentatively attractive for targeting mitochondrial damage. Although direct research on PEVs-mediated mitochondrial rescue remains limited, current evidence suggests that PEVs can modulate diseases associated with mitochondrial dysfunction and potentially enhance mitochondrial health. This review explores the therapeutic potential of PEVs in neurodegenerative and cardiovascular disorders, highlighting their role in restoring mitochondrial health. By examining recent advancements in PEVs research, we aim to shed light on novel strategies for utilizing PEVs as therapeutic agents. Our goal is to underscore the importance of further fundamental and applied research into PEVs-based interventions, as innovative tools for combating a wide range of diseases linked to mitochondrial dysfunction.

## 1 Introduction

Mitochondria are vital organelles responsible for energy production, cellular metabolism, and regulation of apoptosis.^1^ Dysfunctional mitochondria are linked to various diseases, including metabolic syndromes, neurodegenerative and cardiovascular disorders, and autoimmune and age-related diseases.^2^ This connection has fueled interest in mitochondrial-targeted therapies for these illnesses. Among those, extracellular vesicles (EVs), ranging from nanometer to micrometer scale, play a crucial role in intercellular communication by transporting biological materials, including mitochondrial components, between cells.^3^ Therefore, EV’s ability to mediate mitochondrial transfer presents an exciting frontier for biomaterials clinical research.^4^ Their potential to cross biological barriers like the blood-brain barrier (BBB) and deliver targeted therapeutic cargo makes EVs attractive for biotherapeutic applications potentially aiming as restoring mitochondrial dysfunction. While mesenchymal stem cell-derived EVs (MSC-EVs) have been extensively studied, platelet extracellular vesicles (PEVs) offer a less explored yet potentially impactful option for mitochondrial therapies.^5^

Platelets, known for their role in blood clotting, release PEVs upon activation. These vesicles, approximately 0.1 to 1.0 μm in size, carry various molecules, including proteins, RNA, and, in some cases, mitochondrial components or fragmented mitochondrial structures.^6^ Notably, some studies suggest that mitochondrial components within PEVs may retain partial functionality possibly including oxygen consumption and ATP production.^7^ These observations, make the exploration of PEVs for mitochondrial dysfunction. Beyond their therapeutic potential, mitochondria in PEVs influence immune responses by acting as damage-associated molecular patterns (DAMPs), which can activate innate immunity.^8,9^ While this highlights their importance in immunomodulation, it also raises concerns about their potential to contribute to inflammatory conditions, such as autoimmune diseases and transfusion-related adverse events. Previous studies have demonstrated that autoantibodies including antimitochondrial antibodies (AMA), anticardiolipin (aCL), and antimitofusin 1 (anti-MFN1) have been linked to autoimmune diseases such as systemic lupus erythematosus (SLE). These antibodies target key mitochondrial components such as hypomethylated CpG-rich mtDNA and proteins present in both the inner and outer mitochondrial membranes.^10,11^ Additionally, patients with SLE often exhibit elevated levels of EV-associated immune complexes carrying mitochondrial markers like TOMM-20, suggesting that these complexes may play a role in activating the immune system.^10^ Furthermore, repeated administration of allogeneic or xenogeneic EVs has been shown to stimulate the adaptive immune system more, as EVs are known to carry higher levels of major histocompatibility complex (MHC) molecules on their surface, which can trigger immune responses.^12^ This duality underscores the necessity for a deeper understanding of PEVs mitochondria’s biology and functions.

This review assesses the therapeutic potential of PEVs in treating mitochondrial dysfunction. It also addresses challenges such as the need for standardized methods for isolation and characterization and suggests future directions for utilizing PEVs in therapeutic and drug delivery applications targeting diseases involving mitochondrial dysfunction. By integrating existing literature, the review aims to provide a thorough overview of the emerging roles of PEVs in mitochondrial medicine.

## 2 The Multifaceted Role of Mitochondria in Cellular Health

Mitochondria are double-membraned structures central to cellular energy metabolism and signaling. Enclosed by two distinct phospholipid bilayers, they establish compartmentalization critical for their diverse functions. The extensive folding of the inner membrane, the cristae, plays an important role in oxidative phosphorylation (OXPHOS), the metabolic process by which ATP is produced in the mitochondria through the transfer of electrons along the enzyme complexes (I–IV) of the electron transport chain (ETC), which generates a proton gradient that drives ATP synthesis by ATP synthase (Complex V).^1^ Essential metabolic functions like the tricarboxylic acid cycle (TCA cycle), a key pathway in cellular respiration that generates NADH and FADH_2_ for OXPHOS, take place in the matrix, the compartment enclosed by the inner membrane.^13^ The intermembrane space (IMS) between the inner and outer membranes houses proteins that are crucial for mitochondrial cell signaling and apoptosis such as cytochrome C, which also plays a role in the ETC.^14^

### 2.1 Principles of Mitochondrial Bioenergetics

The ETC and the TCA cycle are crucial for the cellular energy production process. Acetyl-CoA, which is produced from pyruvate, fatty acids, and amino acids, is metabolized via the TCA cycle into CO_2_, producing NADH and FADH_2_ that fuels the ETC.^15,16^ The electron transfer cascade is started by Complex I (NADH dehydrogenase) and Complex II (succinate dehydrogenase), which donate electrons to ubiquinone, which then transfers them to Complex III (cytochrome c reductase) and transferred to Complex IV (cytochrome c oxidase) via cytochrome c.^17^ Electron flow drives proton translocation across the inner membrane, generating an electrochemical gradient known as the proton-motive force that powers ATP synthesis via F_1_F_0_-ATP synthase.^18^ The adenine nucleotide translocase (ANT) then converts ATP to cytosolic ADP, further adjusting the potential of the mitochondrial membrane.^19−21^

### 2.2 Mitochondria and Cellular Calcium Dynamics

Mitochondrial Ca^2+^ uptake, which is crucial for proper mitochondrial metabolism, is primarily regulated through the mitochondrial calcium uniporter (MCU).^22,23^ This channel is activated when cytosolic calcium levels rise, facilitating Ca^2^^+^ entry into the mitochondrial matrix. When mitochondrial calcium levels rise, the Na^+^/Ca^2^^+^ exchanger (NCLX) exports excess Ca^2^^+^ from the mitochondrial matrix into the intermembrane space, from where it can diffuse into the cytosol.^24^ However, when mitochondrial Ca^2^^+^ levels exceed 400–500 nM in some cell types, the NCLX becomes overwhelmed, resulting in net Ca^2^^+^ accumulation and subsequent mitochondrial damage.^25^ NCLX activity has a substantial impact on maintaining mitochondrial Ca^2^^+^ balance, preventing Ca^2^^+^ overload that leads to mitochondrial damage and dysfunction.^26^

### 2.3 Mitochondria Dysfunction

**Figure 1:**
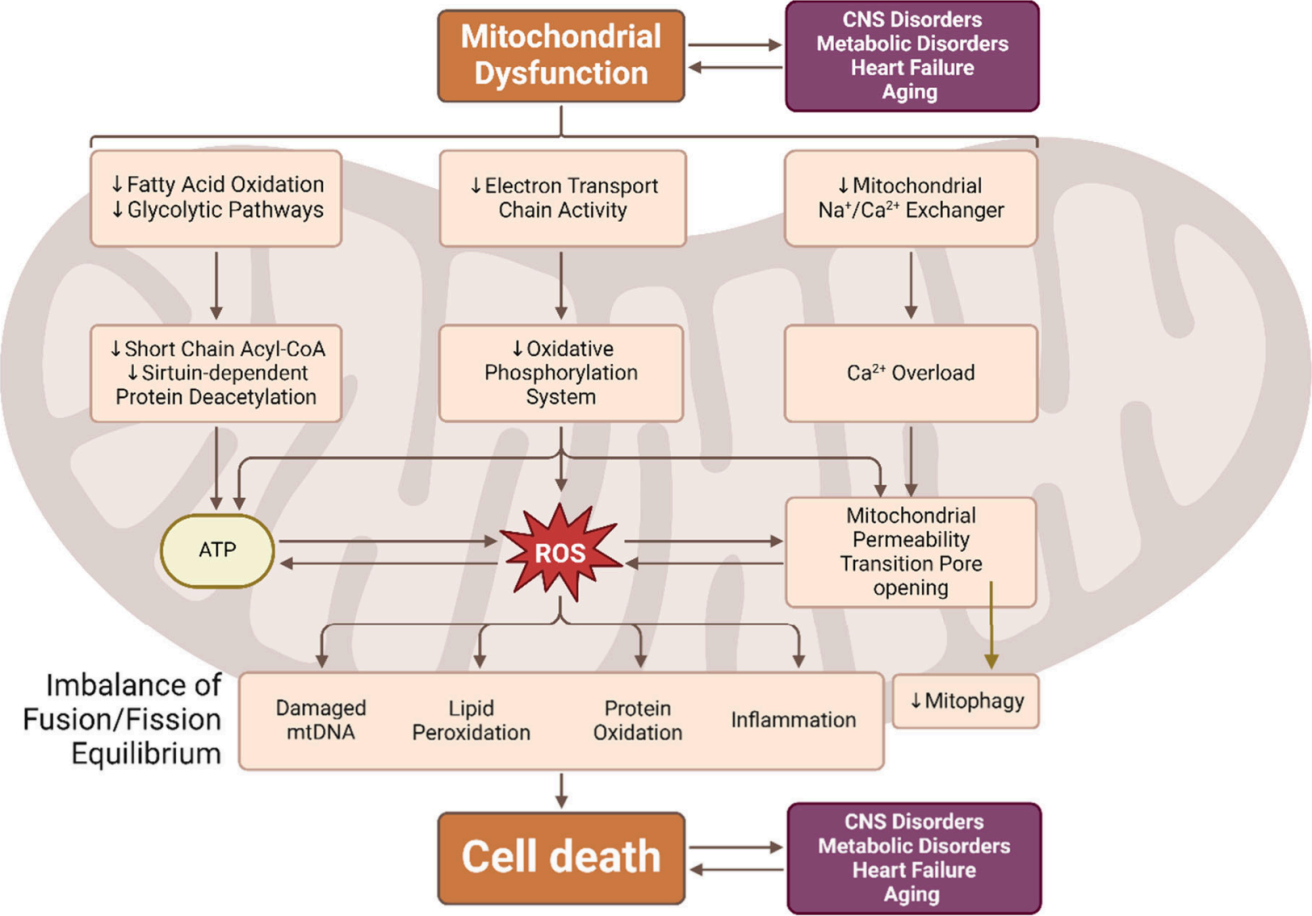
**Pathophysiology of Mitochondria**. Mitochondrial dysfunction leads to increased reactive oxygen species (ROS) production, Ca^2^^+^ overload, reduced ATP production, and opening of the mitochondrial permeability transition pore (MPTP). These events collectively contribute to cellular damage and death. *Created in BioRender*. *Yeh*, *H*. *(2025)*https://BioRender.com/m80h071.

Disruptions in mitochondrial bioenergetics (Figure 1) have been shown to contribute to and result from various diseases, including neurodegenerative disorders Alzheimer’s disease (AD), Parkinson’s disease (PD), amyotrophic lateral sclerosis (ALS), Huntington’s disease (HD) as well as metabolic syndromes, and cardiovascular diseases (CVDs), and muscular degenerative conditions and age-related myopathies (Table 1).^2,27,28^ Common abnormalities include abnormal reactive oxygen species (ROS) production, calcium imbalance, defective mitochondrial dynamics, changes in mitochondrial signaling pathways, and apoptosis dysregulation.^29−33^ Additionally, deficits in OXPHOS, impaired glucose absorption, and poor TCA cycle activity further worsen mitochondrial dysfunction.^30,34^

**Table 1:** Association of Mitochondrial Dysfunction with Diseases^a^^,^^b^

**Target Disease**	**Association with Mitochondrial Dysfunction**
Alzheimer’s Disease (AD)	Impaired mitochondrial bioenergetics, calcium dysregulation, and oxidative stress may trigger Aβ plaque formation and tau hyperphosphorylation, driving neuronal and synaptic loss linked to cognitive decline in AD.^35−39^ Dysfunctional mitochondria further interact with Aβ/tau, accelerating AD progression.^35,40−42^
Parkinson’s Disease (PD)	Mitochondrial dysfunction can lead to dopaminergic neuron loss.^43,44^ Mutations in Parkin, PINK1, DJ-1, LRRK2, α-synuclein genes impair mitophagy and mitochondrial dynamics.^45−47^ Mitochondrial DNA haplogroups mutations and bioenergetic deficits can further contribute to PD progression.^45,48,49^
Traumatic Brain Injury (TBI)	TBI can cause mitochondrial dysfunction that drives secondary brain damage by increasing ROS, oxidative stress, apoptosis, and reducing ATP production.^50−52^
Cerebral Ischemia	Cerebral ischemia impairs mitochondrial ATP production and triggers oxidative stress, apoptosis, and neuronal death.^53,54^ Reperfusion worsens injury via MPTP opening, calcium overload, and ROS generation.^53,55,56^
Atherosclerosis	Oxidative stress and endothelial damage lead to mtDNA damage and oxidative phosphorylation disruption, reducing ATP production and impairing vascular smooth muscle cells.^57−59^
Myocardial Infarction (MI)	MI can disrupt mitochondrial ATP production, causing oxidative stress, ca^2^^+^ overload, and cell death.^60−62^ Like cerebral ischemia, reperfusion causes further damage through MPTP opening, ROS production, and apoptosis.^60,63,64^
Intervertebral Disc Degeneration (IVDD)	Elevated ROS induces oxidative stress, apoptosis, and metabolic imbalance in NP cells.^65−68^
Osteoarthritis (OA)	Mitochondrial dysfunction in OA reduces ATP production, increases ROS, and promotes chondrocyte apoptosis, leading to cartilage degradation.^69,70^ mtDNA haplogroup mutations may also contribute to OA progression.^70−72^
Chronic Wound	Mitochondria dysfunction leads to ROS buildup, causing oxidative stress and inflammation, causing wounds to not heal.^73−75^

a Abbreviations: Aβ: Amyloid-beta; DJ-1: Parkinsonism associated deglycase (PARK7), LRRK2: Leucine-Rich Repeat Kinase 2; ROS: Reactive Oxygen Species; ATP: Adenosine Triphosphate; ROS: Reactive Oxygen Species; mtDNA: mitochondrial DNA; MPTP: mitochondrial Permeability Transition Pore; NP: Nucleus Pulposus.

b Mitochondrial dysfunction not only contributes to the pathophysiology of disease progression but can also result from diseases. Common diseases associated with mitochondrial dysfunction include neurological disorders, cardiovascular diseases, degenerative diseases, and chronic wound formation.

Mitochondrial dysfunctions may lead to long-term mitochondrial damage, irreversible cell death, and the progression of degenerative diseases.^33^ In some cases, it occurs as a consequence rather than the primary cause of pathology, with neuroinflammation and oxidative stress further exacerbating the condition. This interplay, along with proteinopathies, kinetic imbalances, genetic abnormalities, and calcium dysregulation, drives the development of various metabolic and neurodegenerative diseases.^2,27,28^

## 3 Introduction to Extracellular Vesicles

Initially regarded as mere cellular waste disposal systems, EVs are now recognized as crucial mediators for intercellular communication. In 1946, Chargaff and West proposed the existence of ″pro-coagulant platelet-derived particles”. Further electron microscopy studies between the mid-1960s and early 1980s provided additional evidence of submicron structures matching the dimensions of what we know as EVs.^76^ By the early 1980s, EV research had expanded, marked by a notable surge in publications, theories, and debates over nomenclature. Today, research on EVs involves a wide array of experimental techniques, as a review in 2017 already identified a total of 1,742 experiments utilizing 190 different isolation methods and 1,038 protocols.^77^ While no universally accepted method has yet emerged, this methodological diversity and frequent gaps in reporting present challenges for comparing and interpreting findings across studies.

EVs are often classified by size into three main subtypes. The smallest are exosomes (Exo), which originate from the endosomal system and measure approximately 30–100 nm in diameter. Followed by microvesicles (MVs) released from the plasma membrane and measuring approximately 100 nm-1 μm. The largest are apoptotic bodies, which develop from apoptosis and measured over 1 μm.^78^ Regardless of their size, all EVs share key features: they are enclosed by a lipid bilayer, lack the ability to replicate (due to the absence of a functional nucleus), and naturally carry molecular cargo such as proteins, lipids, and nucleic acids often derived from the cytosol and plasma membrane.^78^ EVs are biologically derived, membrane-enclosed vesicles that actively package and transport these biomolecules unlike general passive lipid aggregates that typically arise as byproducts of metabolic processes and often lack the regulated cargo loading.^79,80^ EVs are biologically derived vesicles secreted by cells, naturally carrying a variety of biomolecules, including RNA, proteins, and lipids, with inherent targeting abilities depending on their cellular origin. They are involved in intercellular communication and they possess specific biogenesis and secretion mechanisms, such as the ESCRT (Endosomal Sorting Complex Required for Transport)-dependent and ceramide-mediated pathways.^81^ They have been shown to cross biological barriers, making them a promising vehicle for RNA delivery with minimal immunogenicity. On the other hand, lipid nanoparticles are synthetically engineered particles designed specifically to encapsulate therapeutic payloads, particularly RNA, and are often modified with additional surface ligands to improve targeting and reduce immune recognition. While lipid nanoparticles are commonly used in RNA-based therapies like mRNA vaccines, they can trigger immune responses and face challenges with liver clearance and lysosomal degradation upon cellular uptake. EVs, due to their natural origin, typically have better biocompatibility and stability in circulation, offering a potential advantage over lipid nanoparticles for long-term, repeated administrations. However, both systems must overcome cellular barriers to deliver their therapeutic cargo effectively, with EVs benefiting from their ability to bypass these barriers more efficiently.^82^ Recent advancements in the EV field have led to numerous clinical trials exploring their potential as targeted therapeutics, alongside the clinical success of LNP-based therapies.^83^

EVs carry out diverse and complex biological functions. While the exact cellular pathways (i.e., paracrine, autocrine, and endocrine) EVs involved are not fully characterized, EVs are well-known to participate in intercellular communication. Cells both secrete EVs and internalize them from their surroundings, facilitating an exchange of materials that can induce to molecular changes in recipient cells across different tissues.^84^ Recent findings reveal that exosome biogenesis involves complex mechanisms beyond the conventional ESCRT-dependent pathways, including ceramide-mediated pathways, tetraspanins, and neutral sphingomyelinase 2 (nSmase2). These mechanisms regulate the selective loading of cargo into EVs, including DNA, RNA, and proteins, thereby contributing to the functional diversity of Exo. This growing understanding has spurred interest in EVs as mediators of intercellular communication and as potential biomarkers or therapeutic agents for a range of diseases, including cancer and neurological disorders.^85^

## 4 Extracellular Vesicles and Mitochondrial Dysfunction

EVs have shown promise for both the treatment of mitochondrial dysfunction and as a source of biomarkers for its diagnosis, as they can transport mitochondrial components or mitochondria-derived structures to target cells. Additionally, EVs contain trophic factors that support mitochondrial function and cellular recovery.

### 4.1 Neurological Disorders

The brain has a remarkably high energy demand, with approximately 50% of its total energy production dedicated to powering Na^+^/K^+^-ATPase pumps that maintain the electrochemical gradient necessary for neuronal signaling.^86^ Beyond ATP generation, mitochondria regulate apoptosis and produce ROS.^87^ Since they are the principal site of oxidative phosphorylation,^88^ making them crucial for brain function and survival. Their dysfunction is also strongly linked to neurodegenerative disorders.

Traditional drug delivery for brain disorders faces challenges due to the BBB, which restricts most therapeutics from crossing. Only 5% of small-molecule drugs can penetrate the BBB.^89^ In contrast, EVs appears to have a natural capacity to cross the BBB, making them promising carriers for neurological therapeutics.^90^

Their unique physicochemical properties, including small size, lipid composition, and interactions with endothelial receptors, contribute to their capacity to traverse this biological barrier. The ability of EVs to cross the BBB is influenced by several key factors.^91,92^ Their size, particularly Exo ranging from 30–100 nm, is within the optimal range for transcytosis, whereas larger vesicles such as MVs have reduced penetration efficiency. Most EVs have a neutral to slightly negative surface charge due to their phospholipid membrane composition which allows for interaction with BBB endothelial cells. Additionally, their lipid composition, which includes cholesterol, sphingomyelin, and phosphatidylserine (PS), plays a role in their stability and cellular interactions. The balance of hydrophobicity and hydrophilicity determines whether EVs undergo fusion with membranes, endocytosis, or receptor-mediated uptake. Surface proteins such as tetraspanins (CD9, CD63, CD81), integrins, and heat shock proteins further facilitate interactions with endothelial receptors, promoting EV translocation across the BBB.^93^

Several mechanisms have been proposed for EV uptake by brain endothelial cells. Receptor-mediated transcytosis (RMT) involves interactions with receptors such as LRP1, transferrin receptors, or Intercellular Adhesion Molecule 1 (ICAM-1) , facilitating vesicle internalization and transport across the BBB. Adsorptive-mediated transcytosis (AMT), based on electrostatic interactions, also contributes to EV uptake.^94^ In some cases, EVs directly fuse with endothelial cell membranes via transcytosis, releasing their cargo into the brain through crossing the BBB.^95,96^ Additionally, pathological conditions that compromise BBB integrity, such as inflammatory or tumor-associated stress, can increase the transport of EVs into the brain.^97−100^

EVs have been proposed to transport mitochondrial components across biological barriers, including the BBB, potentially contributing to the delivery of neuroprotective agents.^101^ This suggests that EVs could serve as natural mitochondrial carriers, transferring bioactive mitochondrial proteins, RNA, or even entire mitochondria to recipient cells. EVs may carry mitochondrial components such as cytochrome c oxidase, ATP synthase subunits, which could contribute to mitochondrial function in recipient cells, potentially mitigating neuronal loss and dysfunction.^102^ In addition, ATP synthesis within EVs helps preserve mitochondrial integrity, enhancing therapeutic efficacy. EV-mediated delivery of mitochondrial components and bioactive molecules has been associated with enhanced neuronal function and may support neurogenesis, offering a potential strategy for treating cognitive and neurodegenerative diseases.^103^ Studies using fluorescently labeled EVs suggest that a small proportion reaches the brain, however exact fraction of EVs that successfully cross the BBB is not well quantified and likely depends on administration route, EV surface modifications, and recipient brain conditions.^104^ Further studies are needed to determine the efficiency, specificity, and therapeutic potential of EV-mediated mitochondrial transfer, as well as strategies to enhance EV targeting to the brain through surface modifications or ligand engineering.

In a mouse model of Alzheimer’s disease (APP/PS1 mice), Li et al. demonstrated that neural stem cell-derived small EVs improved mitochondrial function, synaptic activity, and reduced inflammation by increasing mitochondrial biogenesis (peroxisome proliferator-activated receptor gamma coactivator 1-alpha (PGC1α), nuclear respiratory factor 1 (NRF1), nuclear respiratory factor 2 (NRF2)), mitochondrial dynamics (fission 1 protein (Fis1)), and sirtuin 1 (SIRT1) expression, while lowering oxidative stress markers.^105^ Similarly, in a mouse model of ischemic stroke, medium or large EVs from human brain endothelial cells significantly reduced brain infarct size, potentially due to their higher mitochondrial content, which led to enhanced ATP production in recipient cells.^106^

### 4.2 Cardiovascular Diseases

The heart relies heavily on mitochondrial bioenergy to sustain its continuous contractile activity, with oxidative metabolism in mitochondria serving as its primary energy source.^107^ As the main site of cellular respiration and ATP production, mitochondria play a crucial role in cardiac function.^108^ Any disruption in mitochondrial function impairs ATP synthesis, potentially leading to heart failure, cardiomyopathy, and ischemic heart disease due to the heart’s inability to meet its energy demands.^109^ Traditional heart disease treatments, such as pharmacological interventions and surgery, are often costly, invasive, and associated with side effects,^110^ highlighting the need for alternative, targeted therapies like EVs to address mitochondrial dysfunction more precisely.

In the cardiovascular system, intercellular mitochondrial transfer helps maintain cardiac homeostasis under physiological conditions.^111,112^ However, under pathological cardiovascular conditions, cells release EVs that carry mitochondria with impaired function.^103,113^ Holvoet et al. found that these EVs show lower levels of cytochrome C oxidase I and are linked to increased cardiovascular risk and greater inflammation.^113^ Identifying mitochondrial components in EVs could be a promising strategy for diagnosing CVDs. It is reported that patients with coronary artery disease who later experienced new cardiovascular events had lower levels of mitochondrial RNA (MT-COI) in their plasma EVs, indicating that mitochondrial-enriched EVs may serve as potential biomarkers for CVD prediction.^113^ Recent studies also demonstrated the therapeutic potential of cardiac-derived EVs (cEVs). In a murine model of myocardial ischemia-reperfusion injury, cEVs transferred ATP5a1, a key mitochondrial protein, to cardiomyocytes, improving cardiac function and reducing damage.^114^ Additionally, mitochondria-rich EVs from human-induced pluripotent stem cell (hiPSC)-derived cardiomyocytes enhanced ATP production and contractile function both in vitro and in vivo in a mouse model of myocardial infarction.^115^

Interestingly, EVs from diverse cell sources contain bioactive cargo, including mitochondrial and nonmitochondrial components, supporting cardiac repair. Mitochondria-rich EVs from induced pluripotent stem cell-derived cardiomyocytes (iCMs) enhance ATP production and improve contractile function in hypoxia-injured cardiomyocytes.^115^ Hypoxia-conditioned MSC (hMSC)-derived EVs, enriched with PD protein 7 (PARK7), help alleviate myocardial hypertrophy by reducing mitochondrial oxidative stress.^116^ MSC-EVs have also been shown to play a crucial role in cardioprotection and repair by promoting angiogenesis, reducing apoptosis, and preserving mitochondrial membrane potential through miR-19a enrichment.^117^ Additionally, EVs from bone marrow MSCs (BM-MSCs) overexpressing macrophage migration inhibitory factor (MIF) protect against mitochondrial fragmentation and apoptosis in stressed cardiomyocytes.^118^

Zhang et al. also reviewed the indirect regulation of mitochondrial function through EVs under hypoxic conditions, highlighting the overall mechanisms by which EVs impact mitochondrial activity.^119^ Given their ability to deliver functional mitochondria and mitochondrial components, EVs are now being explored as a novel, cell-free therapy for CVD.^103^

## 5 Current Routes of Extracellular Vesicles Administration and Upscaling for Therapeutic Needs

The choice of administration route plays a crucial role in determining the bioavailability, distribution, and efficiency of extracellular vesicle (EV)-based therapies for various diseases. Several delivery methods have been explored, each offering distinct advantages and challenges in targeting specific tissues and organs. Systemic administration, such as intravenous (IV) and intraperitoneal (IP) injection, is widely used due to its ease of use and broad distribution potential.^120,121^ However, EVs administered systemically often face rapid clearance by organs like the liver, spleen, and kidneys, reducing the amount that reaches target tissues, such as the brain or heart.^122^ Oral administration allows targeted accumulation in the small intestine, distinguishing it from intravenous delivery, which predominantly targets the liver and spleen and have been employed for improving joint inflammation and modulating immune responses.^123^ Interestingly, intranasal delivery offers a noninvasive method that allows EVs to bypass the BBB, which improves brain penetration and reduces systemic clearance, making it beneficial for treating neurodegenerative and neuroinflammatory conditions.^124^ These studies show that intranasally administered EVs can efficiently reach key brain regions, such as the hippocampus and cortex, within hours. On the other hand, direct intracerebroventricular injection bypasses the BBB entirely, ensuring maximum EV concentration at the target site, which is crucial for treating diseases like Alzheimer’s or Parkinson’s. However, the invasive nature of the procedure limits its widespread clinical application.^125,126^ Intrathecal (IT) and intracisternal injections offer a less invasive alternative, delivering EVs into the cerebrospinal fluid (CSF) to achieve significant central nervous system distribution without requiring neurosurgical procedures.^127,128^ These routes are particularly useful for targeting diseases affecting the spinal cord, meninges, or periventricular regions of the brain.

In CVD, systemic EV delivery could potentially target heart tissue and blood vessels, though modifications are needed to improve their specificity for endothelial cells and cardiac tissues. Intramuscular injection of EVs can also target heart muscle tissue directly, making it suitable for conditions like heart failure, where EVs could promote repair and regeneration.^129^ Intracoronary injection and intramyocardial is a promising method for delivering EVs directly to the heart, especially after events like myocardial infarction and ischemia-reperfusion injury by promoting tissue repair and regeneration.^130,131^ This method allows targeted delivery to heart tissue, minimizing systemic clearance and improving therapeutic concentration at the site of injury.

To date, comparative evidence on the various routes of administration remains limited, highlighting the need for further research to optimize delivery strategies and therapeutic outcomes.

To enhance targeting specificity, surface modifications such as ligand functionalization can be applied. For example, conjugating EVs with brain-targeting peptides, like transferrin or rabies virus glycoprotein, can facilitate interactions with BBB receptors or ligands binding to endothelial cells or receptors involved in cardiovascular function (e.g., integrins, selectins).^132^ Advanced techniques such as magnetic guidance and ultrasound-based delivery are also being explored to enhance the precision of EV targeting to specific regions of the brain or heart.^3,133,134^ Despite the promising potential of EVs as drug carriers for targeted therapies, challenges remain in optimizing their distribution, enhancing BBB penetration, and minimizing off-target effects. Researchers are focusing on refining administration strategies, improving EV engineering for selective tissue delivery, and advancing upscaling methods to meet clinical demands.

Effective therapeutic doses of EVs vary depending on the disease model, administration route, and desired therapeutic effect. Preclinical studies typically use EV doses ranging from 10^9^ to 10^12^ particles per dose, with higher quantities required for systemic applications.^135^ However, large-scale production remains challenging due to limited yields from conventional isolation methods. To ensure reproducibility and regulatory compliance, EV therapies must adhere to stringent quality control and standardization guidelines, such as accurate quantification through nanoparticle tracking analysis (NTA) or tunable resistive pulse sensing (TRPS), removal of coisolated proteins, lipoproteins, or cell debris to maintain therapeutic efficacy, and verification of cargo integrity (proteins, RNA, lipids) and biological activity across production batches. Additionally, optimized storage conditions, such as cryopreservation at −80 °C or lyophilization, are necessary to maintain EV bioactivity over extended periods.^136^ As research progresses, administration routes, targeting, and upscaling will be vital to unlocking the full therapeutic potential of EVs for a wide range of diseases.

## 6 Platelet Extracellular Vesicles

### 6.1 Introduction to Platelet Extracellular Vesicles

Platelets are essential for hemostasis by forming coagulation plugs. In addition, they contribute to the immune response by releasing signals that recruit immune cells to sites of infection and inflammation.^137^ However, since platelets are confined to the bloodstream, they release PEVs, which, due to their smaller size, can travel beyond the vascular system.^6^

PEVs were first described by Wolf in 1967, who isolated them via high-speed centrifugation and termed them ″platelet dust”.^138^ PEVs are generally classified into two microparticles (MPs) (also known as MVs or ectosomes) (100 nm – 1 μm), which are shed from the plasma membrane, and smaller vesicles (40 – 100 nm), which may originate from the endosomal compartments.^139^ They are naturally released during platelet activation, stress, or apoptosis, with their composition influenced by the specific stimulus triggering their formation.^139,140^

Upon activation, platelets shed microvesicles (PMVs), which externalize anionic phospholipids such as PS on their surface, contributing to coagulation. The PMVs contain PS, phosphatidylethanolamine, and, potentially under some pathophysiological conditions, tissue factor (TF), which can enhance thrombin generation and clot formation.^141,142^ However, the procoagulant activity of PEVs depends on the platelet activation mechanism (e.g., stimulation via ADP or epinephrine), with certain activation pathways generating PEVs that lack procoagulant activity.^6,143^ PEVs isolated from plasma often fail to bind annexin V unless stimulated, suggesting that many circulating PEVs lack surface-exposed procoagulant phospholipids.^143,144^

**Figure 2:**
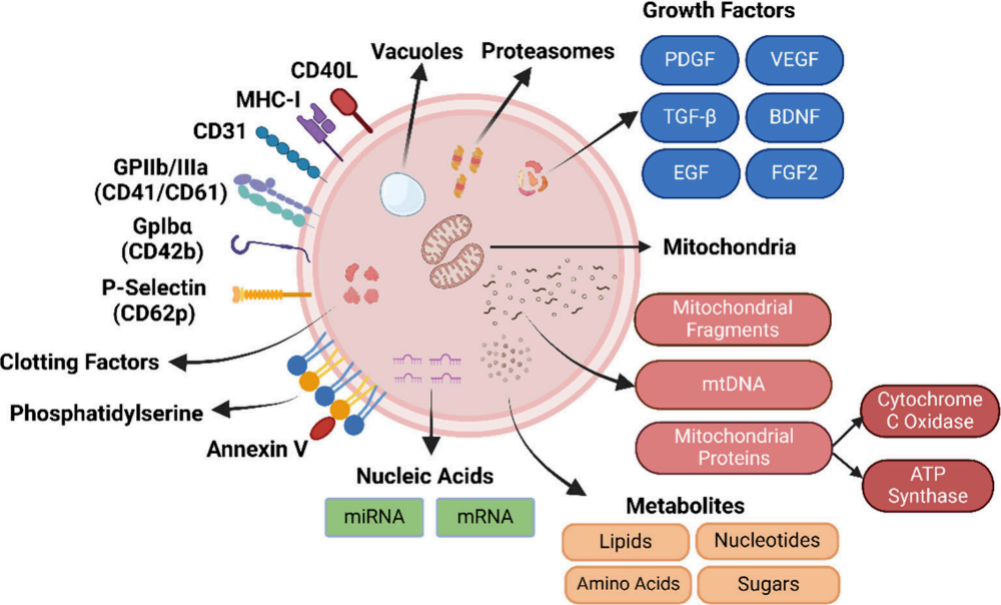
**Molecular Cargo and Surface Markers of Platelet Extracellular Vesicles**. PEVs may carry a diverse array of molecular cargo, including organelles such as vacuoles and mitochondria, nucleic acids like miRNA and mRNA, metabolites, growth factors, and clotting factors. PEVs also express a wide range of surface markers. The specific types of molecular cargo and surface markers present in PEVs are influenced by the physiological conditions they are generated, thereby determining their identity and functional roles.^139,140^*Created in BioRender*. *Yeh*, *H*. *(2025)*https://BioRender.com/p87e893.

PEVs are an abundant, possibly the most prominent type of EVs in circulation, and act as important signaling particles between platelets and other cells, significantly contributing to intracellular communication.^145,146^ PEVs possess the ability to cross various tissue barriers, potentially including the BBB,^6^ but direct experimental evidence still remains limited at the moment. *In vitro* studies have demonstrated that PEVs can internalize into human brain endothelial cells by endocytosis.^147^ Additionally, their surface markers distinguish them from other EVs. Many PEVs express P-selectin (CD62p), integrins, and adhesion receptors (CD63, CD31, and GPIIb/IIIa (CD41/CD61), GpIba (CD42b)) (Figure 2).^140,148^ Furthermore, PEVs contain platelet-derived molecules such as mRNA, cytokines/chemokines, and growth factors (such as Platelet Factor 4 (PF4), Brain-Derived Neurotrophic Factor (BDNF), Platelet-Derived Growth Factor (PDGF), Epidermal Growth Factor (EGF), Basic Fibroblast Growth Factor (bFGF), Transforming Growth Factor Beta (TGF-β), Fibroblast Growth Factor 2 (FGF2), and Vascular Endothelial Growth Factor (VEGF)),^142,149−151^ which promote angiogenesis, tissue regeneration, and immunomodulation, indirectly supporting mitochondrial function by improving mitochondrial oxidative capacity, metabolism, and nutrient supply.^152−154^ PEVs carry a wide variety of molecular cargo, including nucleic acids (mRNA, miRNA), proteins, lipids, and proteasomes that can be transferred to other cells, thus influencing their functions.^155^ This cargo not only influences cellular functions but also plays a crucial role in regulating mitochondrial biogenesis and metabolism by modulating gene expression in recipient cells. For instance, a subset of PEVs has been shown to localize with mitochondria and deliver miRNAs such as miR-24, which modulates mitochondrial gene expression.^156^ This transfer further leads to mitochondrial dysfunction and apoptosis by targeting genes like mt-Nd2 in tumor cells. The same study also demonstrated that under noncancerous conditions, PEVs contribute to mitochondrial homeostasis by mitigating oxidative stress, enhancing mitochondrial biogenesis, and regulating metabolic pathways. This highlights the broader role of PEVs in cellular metabolism, tissue repair, and disease modulation, making them promising candidates for therapeutic strategies targeting mitochondrial dysfunctions. However, there are still unanswered questions in this domain, and a more comprehensive investigation is required to identify the mechanisms through which PEVs contribute to mitochondrial rescue.

### 6.2 Isolation and Detection of Platelet Extracellular Vesicles

There are currently no universal, standardized methods for EV isolation in the laboratory or as a means to develop EV-based therapies. In the case of PEVs, isolation and purification involve separating EVs from not only the non-EV components of the blood, such as blood cells and proteins but also from platelets themselves.^139^ This step remains challenging, because some non-EV components (e.g., lipoproteins) share similar size or density as PEVs, and may be coisolated.^139^ According to the MISEV2023 guideline, non-EV structures like apolipoproteins, albumin, or immunoglobulin can coisolate with blood and plasma-derived EVs.^78,157^ Moreover, platelets themselves share many features with general EVs such as being membrane-bound and often express similar molecular markers such as tetraspanins, complicating their distinction. In the context of hemostasis, platelets are involved in the formation of a hemostatic plug at sites of vascular injury, and their procoagulant activity is closely linked to that of EVs. Both platelets and EVs express PS on their surfaces, which is important for coagulation factor binding and fibrin formation. Given this overlap, the inability to effectively separate platelets from PEVs can impact the interpretation of studies.^158^ However, the PEVs functionality remains intact regardless of the presence of platelet markers, reinforcing their relevance as biomarkers and therapeutic agents as seen in various disease contexts. This reinforces the critical need to establish standardized protocols for the isolation and purity verification of EVs in general and PEVs in particular.

A common approach for isolation is differential centrifugation. This process begins at low speeds to remove cells and debris, followed by higher-speed spins (approximately 14,000–30,000 × g) to isolate larger EVs.^159,160^ A subsequent ultracentrifugation step at ∼100,000 × g is often used to retrieve smaller EVs.^160,161^ It is important to note that, even though ultracentrifugation is widely used, it can potentially alter MV properties and affect their functionality. An alternative approach gaining popularity is size exclusion chromatography (SEC), a relatively gentle technique that helps preserve functionality.^162,163^

The impact of PEVs isolation methods on its purity, composition, functionality, and trophic factor content remains an area of active research. These isolation techniques influence their molecular cargo, including mitochondrial components and bioactive molecules. Though not specifically in PEVs, previous studies have shown that high-speed centrifugation yields EVs enriched in mitochondrial proteins, which can induce early apoptosis in cancer cells, whereas sucrose cushion and ultracentrifugation methods produce EVs enriched in proteins associated with extracellular matrix (ECM) interactions and immune responses.^164^ Additionally, lysosomal inhibition has been found to increase the secretion of mitochondria in large EVs, suggesting that cellular conditions also play a role in determining EV mitochondrial content.^165^ However, further research is necessary to fully clarify these effects and to establish standardized isolation protocols for both EVs and PEVs in therapeutic applications.

Following isolation, thorough biophysical characterization of the isolated PEVs is important to determine their molecular profile. Methods such as NTA, TRPS, and dynamic light scattering (DLS), can provide data on size and particle counts. Notably, PEV particle count can also be influenced by factors such as unknown levels of residual platelets in biobanked samples, platelet activation, and variations in preanalytical processing, including the choice of anticoagulant or serum. Electron microscopy (TEM, cryo-EM) and surface plasma resonance-atomic force microscope (SPRi-AFM) can further confirm PEVs morphology and verify their lipid bilayer structure.^166,167^ Additionally, various functional activity assays are designed specifically to measure PS- and, possibly, TF-expressing PEVs.^168^ Furthermore, it is also common practice to evaluate the surface marker expression, as well as their protein, growth factor, cytokine, and miRNA content, to better characterize the EV cellular origin and function.

## 7 Platelet Extracellular Vesicle Mitochondria

### 7.1 Introduction to Platelet Mitochondria

Due to their anucleate nature, platelets rely heavily on mitochondria to sustain their physiological functions. Each platelet possesses 5–8 mitochondria, supplying energy via glycolysis and aerobic respiration.^169^ Interestingly, platelet mitochondria can shift between glycolysis and aerobic respiration depending on their surroundings.^170^ In addition, they contribute to β-oxidation by supplying acetyl-CoA for ATP generation. They also facilitate anaplerotic reactions, such as glutaminolysis, to replenish TCA cycle intermediates and sustain metabolic function.^171−173^ This metabolic adaptability allows platelets to function under various conditions, including during activation.^172^

The effects of mitochondrial metabolism on platelet function have been studied extensively. Richman et al. highlighted the importance of mitochondrial gene expression by knocking out nuclear-encoded genes for RBPs (ELAC2, PTCD1, or MTIF3), which impaired mitochondrial function, reduced COXII protein, and led to thrombocytopenia and increased bleeding time.^174^ Nayak et al. found that inhibiting pyruvate dehydrogenase kinases (PDKs) shift metabolism from glycolysis to OXPHOS, but excessive OXPHOS impairs activation and thrombi stability.^175^ A recent PDK2 and PDK4 knockout model reinforced these findings, demonstrating that an optimal metabolic balance is essential for regular platelet function.^176^

Platelet mitochondria also participate in mitophagy to remove dysfunctional mitochondria. Given their anucleated nature, platelets require active mechanisms to eliminate damaged mitochondria.^177^ The Parkin/PINK1 pathway is well-known in this context and is upregulated in diabetes mellitus to protect platelets from oxidative stress, with Parkin also potentially interacting with proteins involved in activation and aggregation.^178^ Both Nix (BNIP3L) and FUNDC1 proteins are also receptors involved with platelet mitophagy.^179,180^ Defects in Nix have been linked with a higher risk of thrombosis due to impaired platelet activation.^179^ On the other hand, activation of FUNDC1-mediated mitophagy has been shown to protect cardiac muscle following ischemia/reperfusion (I/R) injury.^180^

Given the multifaceted roles of mitochondria in platelets, the possibility that PEVs contain functional, respiratory-competent mitochondria when released by platelets has been investigated but the extent to which these mitochondria remain fully functional requires further validation. Although current studies are still limited, we highlight some existing literature below. It is important to note that this field still requires further exploration, including whether PEVs harbor intact, functional mitochondria, which type of PEVs yields the most mitochondrial material, and whether isolation techniques can be optimized for consistently yielding functional mitochondria. These questions remain important questions for future investigation.

### 7.2 Presence of Functional Mitochondria in Platelet Extracellular Vesicles

Boudreau et al. observed the presence of platelet microparticles packed with mitochondria (mitoMPs) released from thrombin-activated platelets under transmission electron microscopy (TEM).^181^ MitoMPs are hypothesized to be released during platelet activation because of the proximity of mitochondria to the platelet membrane during the budding process induced by platelet activation.^181^ Brisson et al. further supported this concept, as they observed mitochondria (approximately 300 nm in size) present in large PEVs fragments under cryo-EM. However, mitochondrial presence within PEVs was only occasionally observed in activated platelet samples.^145^ De Paoli et al. observed platelet vesicles containing mitochondria under cryo-TEM, offering a further explanation for this hypothesis.^182^ They propose that, upon platelet activation, cytoskeletal contraction raises hydrostatic pressure against the platelet membrane. As a result, “blistering” of the platelet membrane occurs, causing vesicles encapsulating mitochondria to bud off from the platelet surface.^182−184^

MitoMPs were also found in platelet-free plasma (PFP), derived from platelet concentrates (PCs), stored over 5 days at approximately room temperature (20 °C∼24 °C), suggesting the possibility of finding EV-encapsulated mitochondria even after extended storage.^181^ Marcoux et al. later confirmed these findings by identifying a subset of MPs containing organelles (including mitochondria) under TEM, 7 days after storage under 20 °C∼24 °C.^9^ Although using TEM to confirm the presence of mitochondria encapsulated within PEVs proved more challenging, the introduction of high-sensitivity flow cytometry (hs-FSC) offered stronger evidence for detecting mitochondrial components within PEVs, aided by platelet and mitochondrial markers.^7^ However, more studies are needed to establish whether hs-FSC can reliably determine the absolute concentration of PEVs mitochondria.

Besides detecting the presence of mitochondria in PEVs, determining whether these encapsulated mitochondria remain respiratory competent is another focus. Boudreau et al. demonstrated that mitoMPs are respiratory competent based on their ability to consume oxygen, as measured using a temperature-controlled polarographic oxygen monitoring system.^181^ Additionally, Pelletier et al. conducted functional analysis of PEVs mitochondria, referred to as “mitlets” in their study, utilizing the Seahorse XF analyzer. Oxygen Consumption Rates (OCR) measurements showed distinctive changes following sequential injections of mitochondrial substrates and inhibitors, confirming that all mitochondrial complexes were functional and respiratory-competent. An ATP production assay also demonstrated that mitlets not only contain ATP but generate ATP de novo, indicating that they are respiratory competent even if encapsulated within PEVs.^7^

PEVs bearing mitochondrial components have been shown to enhance the metabolic function of recipient cells, mirroring results observed in stem cell-EVs research.^6^ PEVs may regulate key mitochondrial pathways (SIRT1–PGC1α–TFAM) by maintaining or restoring mitochondrial function.^185^ They also carry miR-24, which regulates apoptosis and mitochondrial dysfunction in tumor cells,^186^ and miR-155–5p, which exacerbates ischemia-reperfusion injury by interfering with cyclophilin D, a regulator of the mitochondrial permeability transition pore.^187^ Additionally, thrombin-derived PEVs contain AIFM1, a mitochondrial protein involved in cell death, suggesting their role in necrosis via mitochondrial pathways.^187^ PEVs containing functional mitochondrial components may modulate signaling pathways in the recipient cell.

In addition to encapsulating mitochondria within PEVs, platelets can release them into the ECM. Boudreau et al. referred to these released mitochondria as “freeMitos,” which are observable under TEM and CSLM. Furthermore, they noted an abundance of freeMitos after 5 days of storage, which accompanied an increase in PFP oxygen consumption.^181^ Marcoux et al. also reported an increased presence of freeMitos released from platelets under TEM in PCs- derived from either buffy coat or apheresis- after 7 days of storage at approximately room temperature.^9^ In another recent study, De Paoli et al. observed freeMitos in apheresis platelets stored under comparable conditions for 7 days.^188^ However, these freeMitos might contain damaged cristae and swollen structures, as well as ongoing mitophagy by the third day of storage. Additionally, PS, a common platelet activation and apoptotic marker, suggests that extended PCs storage is linked to increased platelet activation that increases the release of mitochondria.^188^ Nonetheless, the overall quality of these free mitochondria released by platelets remains uncertain following extended storage. It is also unclear whether an increase in freeMitos is alongside a decrease in PEVs-encapsulated mitochondria. Furthermore, whether encapsulating mitochondria within PEVs can deter or slow down mitophagy is another question that requires further investigation.

### 7.3 Platelet Extracellular Vesicle Mitochondria and Immunity

Other than their therapeutic properties, PEVs mitochondria can also act as DAMPs that cause inflammation via toll-like receptor 9 (TLR9).^8,9^ When PEVs interact with immune cells such as neutrophils, it stimulates the production of lipid mediators like leukotriene B4 (LTB4) and enzymes such as sPLA2-IIA, which then trigger inflammation.^181^ Importantly, sPLA2-IIA not only enhances neutrophil extracellular trap (NET) formation,^181^ but after interaction with PEVs, also leads to the generation of 12(S)-HETE, a critical eicosanoid functioning as a lipid mediator. 12(S)-HETE facilitates the internalization of platelet microparticles (PMPs) and their encapsulated cargo, including mitochondria, transcription factors, and miRNA, into neutrophils via the BLT2 receptor.

Since mitochondria can act as DAMPs, mitoMPs have been related to autoimmune diseases.^189,190^ Increased levels of sPLA2-IIA have been detected in the synovial fluid of rheumatoid arthritis (RA) patients, contributing to disease progression.^189^ Melki et al. have also demonstrated a relationship between PEVs mitochondria and SLE, suggesting that mitochondria may activate inflammatory pathways, thereby promoting SLE when internalized by immune cells. More importantly, Melki et al. noted that the majority of mtDNA integrated into immune cells in SLE patients was previously encapsulated, suggesting that it may have originated from PEVs.^190^ sPLA2-IIA has also been shown to influence internalizing PEVs and their contents into immune cells, exacerbating inflammation.^189^

PEVs and PEVs mitochondria health are critical for transfusion safety, as extended platelet storage leads to platelet storage lesions (PSLs), which increase the release of PEVs containing mitochondria and mitochondrial DAMPs, such as mtDNA and formylated peptides.^191,192^ These mitochondrial DAMPs activate innate immune pathways, including TLR9 and formyl peptide receptors (FPRs), triggering inflammatory cascades that activate leukocytes.^9,191,193^ These immune activations are associated with transfusion-related acute lung injury (TRALI), although there is insufficient evidence to conclude that elevated PEVs levels directly cause TRALI.^9,193−195^

Interestingly, PEVs also contain immune molecules such as CD40L, proteasome-related proteins, and MHC-I, which can activate the adaptive immune response.^196^ Unlike platelets which are confined to the bloodstream, PEVs can travel through the lymphatic system and contributing to immunosurveillance and antigen presentation.^6,196^ This underscores the role that PEVs play in immunomodulation and raises the potential that PEVs could be utilized as therapeutic agents for related disorders.

## 8 Platelet Extracellular Vesiclesto Treat Mitochondrial Dysfunction

### 8.1 Platelet Extracellular Vesicles as Therapeutics for Mitochondrial Dysfunction

Due to their diverse physiological properties, PEVs are suspected to not only contribute to the functional role of platelets but also considered for being therapeutic agents or stand-alone treatments for regenerative medicine.^124,197,198^ Importantly, although most studies utilizing PEVs as therapeutic agents have not directly examined their effects on mitochondria, the observed therapeutic benefits suggest that PEVs may play a role in modulating these diseases. This underscores the critical need for additional research to examine how PEVs influence mitochondrial function, contribute to disease progression in conditions associated with mitochondrial dysfunction, and act therapeutically to restore dysfunctional mitochondria in these disorders (Table 2).

**Table 2:** Effects of Platelet Extracellular Vesicles Treatment on Diseases Associated with Mitochondrial Dysfunction^a^^,^^b^

**Target Disease**	**PEVs Effect**	**Reference**
Parkinson’s Disease (PD)	1)Human PEVs from PCs protected dopaminergic neurons from ferroptosis 2) Intranasal administration of human PEVs preserved dopaminergic neurons in a mice PD model, leading to improved motor functions	(208)
Traumatic Brain Injury (TBI)	Intranasal delivery of human PEVs from PCs reduced inflammation in a TBI mouse model	(208)
Cerebral Ischemia	1) Topical application of human PMPs using gelfoam onto adult hypersensitive rats undergone PMCAO showed neurogenesis, angiogenesis, and neuronal differentiation near infarct border 2) Dose-dependent improvement in neurological function, as behavioral recovery observed around day 20 poststroke	(225)
	1) PMPs from healthy RIPC rats reduced the infarct size after transfusion into MCAO rats, confirmed by fMRI and 2–3–5-TTC staining 2) Behavioral Testing (MNSS) showed no significance after 24 h or over the following 9 days	(226)
	Human PMPs treated on neurosphere cultures of E13.5 mice neural stem cells: 1) Increased neurosphere size 2) Reduced cell mortality 3) Promote Neuronal Differentiation 4) Upregulated pERK and pAkt phosphorylation	(199)
	1) Treatment of human PEVs from SCPL, PPL, and HSCPL on SH-SY5Y neuroblastoma cells showed neurorestoration 2) Human PEVs from SCPL enhanced synaptic formation in primary cortical neurons	(163)
Atherosclerosis	Human plaque explants treated with human PEVs generated from monocyte-platelet aggregates stimulated with Iloprost and TNF were able to 1) Reduce pro-inflammatory cytokines 2) Decrease endothelial activation by decreasing ICAM-1 and VCAM-1 expression 3) Increase anti-inflammatory proteins like Gelsolin and Annexin A1	(213)
	PEVs that carry miR-34c-5p delivered in vitro to HCECs and in vivo to ApoE-KO mice reduce inflammation by downregulating PODXL gene thus inactivating the P38 MAPK pathway.	(216)
	1) CACs from atherosclerotic patients treated with PMPs shown to Secrete RANTES to promote adhesion 2) Rats with hindlimb ischemia injected intravenously with PMPs demonstrated greater neovascularization	(177)
Myocardial Infarction (MI)	Human PMPs were delivered both in vitro using the rat aortic ring model and in vivo into chronic ischemic rats, promoting angiogenesis and revascularization.	(214)
	I/R C57BL/6J mice injected percutaneously with human PEVs from SCPL demonstrated: 1) Enhanced cardiac function 2) Reduced infarct size 3) Increased angiogenesis 4) Increased M2 macrophage differentiation	(217)
Intervertebral Disc Degeneration (IVDD)	1) Human PEVs slowed down disc degeneration (DHI confirmed by MRI and micro-CT) 2) NP tissue structure was preserved, and ROS was decreased 3) Restored mitochondrial dysfunction through SIRT1–PGC1α–TFAM pathway	(68)
	Intradiscal injection of FG@PEVS into IVDD rat model: 1) Suppressed pyroptosis (reduced NLRP3 inflammasome activation) 2) Reduced inflammation (decreased IL-1β and TNF) 3) Restored fatty acid metabolism 4) Inhibited ECM degradation	(227)
Osteoarthritis (OA)	Human platelet exosomes were treated to human primary chondrocytes, and intra-articularly injected into OA mice models, showing: 1) enhanced chondrocyte proliferation + migration 2) Reduced OA progression 3) Reduced cartilage degeneration and inflammation 4) Improve cartilage regeneration	(228)
	Human PEVs and C-EVs treatment were given ex vivo in an OA-induced model using human cartilage explants. PEVs treatment group demonstrated higher collagen and DNA content, but no difference in GAG levels.	(218)
	Monosodium iodoacetate-induced OA rats were treated with Human PEVs and C-EVs. PEVs treatment: 1) Better promoted cartilage regeneration 2) Presented better subchondral bone structure 3) Demonstrated better OARSI scores in female mice	(219)
Diabetic Wounds	1) GelAlg@rGO hydrogel loaded PEVs has shown to improve cell migration, reduce inflammation, and promote wound healing and angiogenesis in diabetic rat models 2) When it was exposed to NIR light further accelerated the wound healing process	(223)
	PAH_0.24_G_37_ hydrogel loaded with PEVs (PAH_0.24_G_37_@PEVs) to treat an SDZ-induced diabetic wound rat model showed: 1) full wound closure after 14 days 2) M2 differentiation (anti-inflammatory) 3) angiogenesis 4) follicle activation. It also showed antioxidant properties, which are suggested to combat ROS buildup caused by mitochondrial dysfunction in chronic diabetic wounds.	(222)
Tendon Degeneration & TSPC Senescence	PL-Exos protect TSPCs from ferroptosis and senescence by activating the AMPK/Nrf2/GPX4 pathway, reducing lipid peroxidation, and preserving proliferation. In rat rotator cuff tear model, PL-Exos enhance tendon-bone healing and improve mechanical strength in vivo, highlighting their therapeutic potential for tendon degeneration.	(220)
Leukemia	Platelet lysates promote mitochondrial uncoupling in leukemia cell lines and primary CD^34+^ leukemic blasts, reducing oxidative stress and increasing resistance to mitochondria-targeted apoptosis evidenced by reduced membrane potential (ΔΨM), transiently increased oxygen consumption, and decreased superoxide levels.	(224)

a Abbreviations: PD: Parkinson’s disease; PEVs: Platelet extracellular vesicles; PCs: Platelet concentrates; TBI: Traumatic brain injury; PMPs: Platelet microparticles; PMCAO: permanent middle cerebral artery occlusion; RIPC: ischemia–reperfusion preconditioning; fMRI: functional magnetic resonance imaging; TTC: Triphenyl tetrazolium chloride; MNSS: Modified Neurological Severity Scores; pERK: Phosphorylated Extracellular Signal-Regulated Kinase; pAkt: Phosphorylated Protein Kinase B (Akt); SCPL: Serum-converted platelet lysates; PPL: platelet pellet lysates, HSCPL: Heat-treated serum-converted platelet lysates; TNF: Tumor Necrosis Factor; ICAM-1: Intercellular Adhesion Molecule 1; VCAM-1: Vascular Cell Adhesion Molecule 1; HCECs: Human Coronary Artery Endothelial Cells; ApoE-KO: Apolipoprotein E Knockout PODXL: Podocalyxin; CACs: Circulating Angiogenic Cells; RANTES: Regulated on Activation, Normal T Cell Expressed and Secreted (also known as C–C Motif Chemokine Ligand 5); DHI: Disc Height Index; CT: Computed Tomography; ROS: Reactive Oxygen Species; SIRT1: Sirtuin 1; PGC-1α: Peroxisome Proliferator-Activated Receptor Gamma Coactivator 1-Alpha; TFAM: Mitochondrial Transcription Factor A; IVDD: Intervertebral Disc Degeneration; NP – Nucleus Pulposus; NLRP3: NOD-, LRR-, and Pyrin Domain-Containing Protein 3 FG@PEVS: Functionalized Graphene-Loaded Platelet Extracellular Vesicles; IL-1β: Interleukin-1 Beta; ECM: Extracellular Matrix; OA: Osteoarthritis; C-EV: umbilical cord mesenchymal stem cells; DNA: Deoxyribonucleic Acid; GAG: Glycosaminoglycans; OARSI: Osteoarthritis Research Society International; GelAlg@rGO: gelatin-alginate hydrogel loaded with reduced graphene oxide; NIR: Near infrared; PAH_0.24_G_37_: Poly(allylamine hydrochloride) Hydrogel; TSPCs: Tendon Stem/Progenitor Cells; AMPk: AMP-Activated Protein Kinase (Energy Homeostasis Regulator); Nrf2 – Nuclear Factor Erythroid 2-Related Factor 2; GPX4 – Glutathione Peroxidase 4; CD34^+^: Cluster of Differentiation 34^+^.

b The table highlights the effects of PEVs treatment across various disease categories, including neurological disorders, cardiovascular diseases, degenerative diseases, and chronic wounds.

#### 8.1.1 Neurological Disorders

One of the early studies on investigating PEVs therapeutic effects neurologically was conducted by Hayon et al. They administered human PMPs to mouse neural stem cells, resulting in increased neurogenesis and enhanced differentiation via the phosphorylated protein kinase B (Akt) and extracellular signal-regulated kinase (ERK) signaling pathways.^199^ Though not mentioned directly in this study, phosphorylated Akt and ERK have previously been shown to exert antiapoptotic effects by phosphorylating and inactivating the BCL2-associated death promoter (BAD), a pro-apoptotic protein that is activated by ROS accumulation resulting from mitochondrial dysfunction in cerebral ischemia.^87^ Importantly, proper regulation of phosphorylated Akt levels has been linked to improved outcomes in neurodegenerative diseases associated with mitochondrial dysfunction, such as PD and AD, by enhancing neuronal survival under oxidative stress and regulating mitophagy.^200^ However, further studies are needed to investigate the underlying cellular mechanisms.

Hayon et al. also noted that combining PMP with trophic factors such as BDNF, PDGF, VEGF, and PF4 can also enhance their efficacy in treating cerebral ischemia.^199^ This aligns with existing literature that established the therapeutic association of these trophic factors with neurological disorders caused by mitochondrial dysfunction.^201^ BDNF can regulate mitochondrial function through the MEK/BCL2 pathway, which in turn prevents neuronal apoptosis and preserves synaptic plasticity.^201−205^ PDGF provides neuroprotective effects by reducing oxidative stress,^202^ regulating the glycosylation of mitochondrial ceramide to maintain proper mitochondrial function,^203^ and even increasing average mitochondrial size.^204^ EGF has been shown to promote neuroprotection by activating key survival signaling pathways, such as phosphoinositide 3-kinase (PI3K)/Akt and mitogen-activated protein kinase kinase (MEK)/ERK1/2, and by inhibiting caspase-dependent apoptosis in neurons.^205^ PF4, released upon platelet activation, has also been shown to promote neurogenesis and neuronal proliferation in the dentate gyrus in the hippocampus.^206^ Recently, PF4 has been identified as a pro-youthful factor that alleviates age-related neuroinflammation, promotes synaptic plasticity, and improves hippocampal-dependent learning and memory in aged mice. The therapeutic effects of PF4 are partially mediated through the CXCR3 receptor, which is involved in the cellular and cognitive benefits observed in the aged brain. Additionally, PF4 appears to rejuvenate the aging peripheral immune system by reducing pro-aging immune factors, thus contributing to the restoration of cognitive function. These findings suggest that PF4 could serve as a potential therapeutic target for counteracting inflammation and cognitive decline in aging, with possible broader implications for neurodegenerative diseases like Alzheimer’s.^207^

Although the therapeutic benefits of these trophic factors in addressing mitochondrial dysfunction in neurological disorders have been established, the specific effects of those released from PEVs on mitochondrial dysfunction still require further investigation. However, it is conceivable that these trophic factors will ultimately have a therapeutic role to play.

Recently, our group demonstrated that PEVs isolated from human platelet lysates exhibit neurorestorative and neuroprotective effects on SH-SY5Y human neuroblastoma cells.^163^ Furthermore, PEVs from serum-converted platelet lysates (SCPLs) even promoted synaptic formation in primary cortical neurons.^163^ In PD cell and mouse models, intranasal administration of human PEVs from human platelet concentrates (PCs) provided neuroprotection to dopaminergic neurons by preventing ferroptosis and led to improved motor functions.^208^ In TBI mouse models, these same PEVs also reduced brain inflammation.^208^ PEVs were found to contain growth factors like BDNF, PDGF, VEGF, and PF4.^208^ In addition, proteomic analysis identified antioxidant enzymes, including superoxide dismutase (SOD) and glutathione peroxidases (GPXs), which help alleviate ROS buildup from dysfunctional mitochondria.^208^ Together, these results indicate that PEVs exert neuroprotective effects and may serve as promising therapeutic agents for neurological disorders. Interestingly, PEVs have been investigated as diagnostic biomarkers for neurodegenerative diseases such as AD and PD.^209,210^ However, further studies are needed to validate their effectiveness, as PEVs have been suggested to be unsuitable as diagnostic biomarkers for Huntington’s disease (HD).^211^

Overall, while previous studies have shown that PEVs and their encapsulated trophic factors can be used to treat neurological disorders, additional research is necessary to specifically address the mitochondrial dysfunctions underlying these conditions. This is important for establishing a direct link between PEVs and their effectiveness in restoring normal mitochondrial function in neurological diseases.

#### 8.1.2 Cardiovascular Diseases

Compared to the nervous system, the pathophysiological effects of PEVs have been more extensively explored in the cardiovascular system. However, findings remain mixed, indicating that PEVs may serve as a double-edged sword in the context of CVD.^212^ This dual nature primarily stems from PEVs’ involvement in the immune system, where they can act as pro-inflammatory agents that exacerbate CVD progression.^96,212,213^ Research has shown that EVs released from platelet-monocyte aggregates in response to tumor necrosis factor (TNF) stimulation promote inflammation, contributing to the formation of atherosclerotic plaques and carotid artery disease (CAD).^213^ Interestingly, the combination of TNF with anti-inflammatory agents like iloprost reduces the production of pro-inflammatory cytokines and decreases endothelial activation markers such as ICAM-1 and Vascular Cell Adhesion Molecule 1 (VCAM-1), which are crucial for plaque development.^213^ These observations suggest that PEVs play a significant role in mediating inflammation that drives CVD and that modulating platelet activation can alter PEVs composition to reduce their pro-inflammatory effects, presenting potential therapeutic opportunities for CVD. Several studies have demonstrated the cardioprotective effects of PEVs preclinically in CVD. In 2005, Brill et al. reported the pro-angiogenic role of platelet-derived microparticles (PMPs) both in vitro and in vivo, driven by VEGF, bFGF, and PDGF through PI3-kinase, Src kinase, and ERK signaling pathways. Notably, these pathways also enhance oxidative phosphorylation, mitigate mitochondrial oxidative stress, and prevent apoptosis. This study demonstrated that human PMP-induced angiogenesis enhances endothelial cell invasion and vessel formation, comparable to VEGF/FGF stimulation in ischemic myocardium, thereby promoting neovascularization and highlighting their potential in therapeutic revascularization. Given that VEGF is a key regulator of mitochondrial biogenesis and PDGF supports mitochondrial integrity and survival under stress, PMP-driven angiogenesis may also contribute to mitochondrial rescue in ischemic conditions.^214^ Similarly, Ohtsuka et al. observed that PMPS release RANTES (CCL5), which binds to CCR1, CCR3, and CCR5 receptors on circulating angiogenic cells (CACs), enhancing their adhesion to endothelial cells and promoting neovascularization in ischemic limbs. In vivo, PMP-CACs significantly enhance blood flow recovery and capillary density in ischemic limbs compared to CACs alone, highlighting their therapeutic potential in restoring vascularization. Building on these findings, the release of bioactive factors, including CCL5 and angiogenic cytokines such as VEGF, bFGF, and PDGF from PMPs suggests that it may play a dual role in vascular and mitochondrial rescue on ischemic tissues.^215^ Furthermore, another study highlighted the anti-inflammatory role of microRNA-34c-5p (miR-34c-5p) in PEVs by targeting podocalyxin-like protein 1 (PODXL) and inhibiting the p38 mitogen-activated protein kinase (p38 MAPK) pathway in coronary artery endothelial cells and apolipoprotein E knockout (ApoE-KO) mice.^216^ The downregulation of p38 MAPK signaling, which is also known to modulate mitochondrial oxidative stress and apoptosis, further supports the notion that PEVs may contribute to mitochondrial homeostasis in endothelial cells and reduce oxidative damage associated with atherosclerotic conditions. More recently, Livkisa et al. demonstrated that human PEVs derived from SCPL enhanced cardiac function, promoted angiogenesis, and reduced infarct regions in ischemia/reperfusion (I/R) mouse models.^217^ The cardioprotective microRNAs and trophic factors carried by these PEVs may support mitochondrial protection and enhance myocardial regeneration and repair, given mitochondria’s essential role in cardiomyocyte survival and response to ischemic injury. While these findings highlight the potential of PEVs as therapeutic agents for CVD, there is currently no direct evidence that they restore mitochondrial integrity or function. Further research is therefore needed to clarify the specific mito-protective effects of PEVs and to determine the optimal conditions for their clinical use. In addition, it should be kept in mind that PEVs may exert both protective and potentially pro-thrombotic effects in CVD, depending on their composition and activation state.

#### 8.1.3 Other Diseases

Outside of the nervous and cardiovascular systems, PEVs have also shown promise in treating degenerative diseases, including osteoarthritis (OA) and intervertebral disc degeneration (IVDD).^68^ An interesting study by Forteza-Genestra et al. compared the efficacy of PEVs with human umbilical cord MSC-derived EVs (C-EVs) in an OA-induced model using human cartilage explants.^218^ They found that the PEVs treatment group not only exhibited higher collagen and DNA levels but also contained higher glycosaminoglycans (GAG) levels, which are key factors for ECM generation. The authors suggest that the enhanced effect of PEVs compared to these C-EVs is likely due to bioactive cargoes encapsulated within the PEVs. Additionally, they compared treatments in Monosodium iodoacetate-induced OA rats, which showed that the PEVs treatment group had better cartilage repair, lower inflammatory and synovitis levels, and improved OARSI scores in female mice.^219^ While these studies do not directly address mitochondrial dysfunction, the improved uptake of PEVs by chondrocytes suggests a potential to combat chondrocyte apoptosis induced by mitochondrial dysfunction. Additionally, their anti-inflammatory effects suggest the possibility of combating ROS buildup associated with defective mitochondria. However, since these studies did not focus on PEVs’ effects on mitochondrial dysfunction in OA, this remains an area for further research to explore potential direct therapeutic benefits. Therefore, a direct therapeutic association between PEVs and mitochondrial dysfunction cannot be made until further investigation.

Nevertheless, Chen et al. recently explored the role of platelet-derived Exo (P-Exo) in ferroptosis, an iron-dependent cell death characterized by lipid peroxide accumulation that disrupts mitochondrial function and accelerates aging. Ferroptosis-induced mitochondrial damage was evident in aged tendon stem/progenitor cells (TSPCs), as TEM revealed mitochondrial vacuolization with irregular or missing cristae. This damage contributed to increased oxidative stress and cellular senescence. However, P-Exo alleviated TSPC senescence by activating the AMP-activated protein kinase (AMPK)/nuclear factor erythroid 2–related factor 2 (Nrf2)/GPX4 pathway. Since AMPK activation enhances mitochondrial biogenesis and protects against oxidative stress, upregulation of GPX4 by PL-Exos reduced MDA levels and mitochondrial ROS, preserving mitochondrial integrity and overall TSPC health.^220^ These findings suggest the therapeutic effects of platelet biomaterials in mitigating joint injury from inflammation, oxidative stress, and ferroptosis-induced mitochondrial damage. However, further investigation is needed to directly assess mitochondrial respiration, membrane potential, and dynamics in response to PEVs treatment.

In IVDD models, Dai et al. highlighted the therapeutic potential of PEVs in addressing mitochondrial dysfunction, a key factor that increases ROS production and inflammation in intervertebral disc degeneration.^68^ Pretreatment with PEVs mitigated H_2_O_2_-induced oxidative damage in nucleus pulposus (NP) cells and rat IVDD models by restoring mitochondrial function, reducing oxidative stress, and re-establishing cellular metabolism. Mechanistically, PEVs may carry bioactive molecules that could influence mitochondrial pathways such as SIRT1–PGC1α–mitochondrial transcription factor A (TFAM), which is essential for mitochondrial biogenesis and repair but direct evidence remains preliminary.^68,221^ This could position PEVs as a novel, mitochondria-targeted therapy, as proteomic analysis revealed that PEVs contain mitochondrial proteins like those found in platelets, including components involved in electron transport and ATP synthesis. Additionally, PEVs were enriched in mitochondrial structures such as the inner and outer membranes, matrix, and intermembrane space, suggesting they may help replenish damaged mitochondrial components in recipient cells. Importantly, the presence of SIRT1 (P48047) in PEVs was significant, as SIRT1 is known to play a crucial role in mitochondrial protection by regulating oxidative stress, metabolism, and mitochondrial biogenesis. The data suggests that after PEVs are taken up by recipient nucleus pulposus (NP) cells, they may restore mitochondrial function by providing mitochondrial proteins and supporting metabolic processes. This implies that PEVs could serve as mitochondrial delivering key factors to rejuvenate impaired mitochondria and improve cellular energy balance in mitochondrial dysfunction-related diseases. However, further research is needed to clarify whether their effects exactly arise from direct mitochondrial transfer or indirect signaling pathways.

Back et al. also discussed the concept that PEVs could be used to treat IVDD, and suggested that PEVs offer several therapeutic benefits, including the delivery of platelet-derived antioxidant enzymes, such as SOD, catalase, and GPX-4, which exhibit anti-inflammatory effects and reduce oxidative stress caused by mitochondrial dysfunction.^222^ Additionally, these PEVs loaded with hydrogels have also shown promise in wound healing, as gelatin or fibrin-based hydrogels can stimulate diabetic wound healing, angiogenesis, and reduction in inflammation.^222,223^ The antioxidant properties of PEVs have been highlighted to combat ROS buildup caused by mitochondrial dysfunction in chronic wounds. These all suggested its potential usage to utilize these PEVs as treatments to target diseases faced by the growing aging population worldwide.

Besides this, it has also been reported that platelet-derived components enhance leukemia cell survival by causing mitochondrial uncoupling, reducing oxidative stress, and increasing resistance to apoptosis. Leukemic cells exposed to these components exhibited lower triglyceride levels and mitochondrial membrane potential along with increased oxygen consumption, indicating a metabolic shift that supports cell survival. In addition, platelet components inhibited Bax oligomerization and reduced both basal and rotenone-induced superoxide levels, which further decreased mitochondrial dysfunction and apoptosis.^224^ Although this protective effect makes leukemia cells more resilient, it could hinder cancer therapies that target mitochondria.

### 8.2 Platelet Extracellular Vesicles Mitochondria to Treat Mitochondrial Dysfunction

Given some emerging evidence that PEVs may contain respiratory-competent, functional mitochondria,^7,181^ the potential of using PEVs-encapsulated mitochondrial transplantation to target mitochondrial dysfunction emerges as an intriguing subject for further investigation Although some studies have examined the efficacy of platelet mitochondrial transplantation,^229−233^ the amount of research remains limited. Even fewer studies have characterized the efficacy of transplantation of mitochondria encapsulated within PEVs.

Pelletier et al. were able to observe an improvement in oxygen consumption and ATP production following PEVs mitochondria internalization into neutrophils. Additionally, a drop in oxygen consumption in neutrophils was observed when the mitochondria had been inactivated beforehand, suggesting that respiratory competence within PEVs-encapsulated mitochondria is important for their therapeutic potential.^7^ However, for PEVs mitochondria to be considered therapeutic for transplantation, there needs to be studies that consistently demonstrate: 1. The presence of whole, intact, and respiratory-competent mitochondria within PEVs and 2. The therapeutic effectiveness on the recipient cell following PEVs or PEVs mitochondria treatment.

It is important to note that for mitochondria transplantation and PEV mitochondrial delivery to become effective therapies, it is crucial to use consistent standards in naming and characterizing the mitochondria involved. Based on the recent guidelines of the International Committee on Mitochondria Transfer and Transplantation Nomenclature (ICMTTN), studies should report five main details: the mitochondria’s origin, the method used for their isolation, their size distribution, whether they are delivered as free mitochondria or EV-encapsulated, and whether any modifications were applied following isolation.^234^ Furthermore, if feasible, evaluating metrics such as membrane integrity and respiratory function can further improve reproducibility.^234^ Detailed reporting enables comparisons between studies and helps establish and refine clinical protocols for mitochondrial therapies.

### 8.3 Platelet Extracellular Vesicles as a Drug Carrier to Treat Mitochondrial Dysfunction

Given the limited literature on using PEVs specifically for targeting mitochondrial dysfunction, we propose leveraging existing literature on drug-loaded EVs and PEVs that modulate mitochondrial functions. This approach serves as a foundation for considering PEVs as potential drug carriers for mitochondrial dysfunction. Preclinical studies have reported that a wide range of drug treatments targeting mitochondrial dysfunction can be delivered via EVs or PEVs. These include antioxidants that reduce mitochondrial ROS production,^235^ Bcl-2 family members that intervene in the mitochondrial apoptotic pathway,^236^ and doxorubicin (DOX), a well-known anticancer drug that induces oxidative stress and activates mitophagy.^237,238^ EVs and PEVs have been shown in cellular and animal models to effectively deliver DOX to target cancer cells.^239−241^ Additionally, berberine, an anti-inflammatory alkaloid that modulates the ROS-mTOR pathway, has been shown to significantly reduce inflammation and swelling in rheumatoid arthritis models.^242^ Other mitochondrial-targeting agents, such as curcumin, siRNA, miRNA, catalase, and GATA Binding Protein 4 (GATA-4), have also been successfully delivered by EVs or PEVs.^117,156,243−245^

For drug loading onto PEVs, several studies demonstrated the potential of utilizing them as drug carriers. Wu et al. incubated PEVs with DOX for 24 h at 37 °C and purified them by SEC. They found that postloading DOX resulted in larger, more effective vesicles compared to preloading into platelets, while surface markers remained unchanged, retaining targeting capacity toward MDA-MB-231 breast cancer cells.^246^ Similarly, Li et al. incorporated Angiopep-2 (ANG) into the membranes of Exo from human mesenchymal stem cells (hMSCs) and electroporated them with siRNA targeting glutathione peroxidase 4 (si-GPX4).^244^ This platform, featuring a Fe_3_O_4_ core and a CD63 antibody-conjugated mesoporous silica shell, has been shown to enhance delivery targeting capabilities. Ma et al. incubated platelets with MCC950, a selective NLRP3 inflammasome inhibitor, at 4 °C for 12 h and then activated platelets as well as centrifuged to obtain MCC950-PEVs. They reported that approximately 86.9% of MCC950 was released from PEVs within 48 h, and intravenous injection of these PEVs in ApoE-KO mice significantly reduced inflammatory cytokines such as interleukin-1 beta (IL-1β) and TNF.^247^

While extensive research exists on drug delivery systems targeting mitochondrial dysfunction and specifically on the use of PEVs as drug carriers, further research is required to characterize whether PEVs can be effectively and efficiently utilized for treating mitochondrial dysfunction.

## 9 Current Challenges and Future Perspectives

Mitochondrial health has recently become a focus of research due to its established connections to various diseases, including neurological, cardiovascular, and degenerative disorders. While previous literature has highlighted the role of EVs in mitigating mitochondrial dysfunction, only a limited number have specifically examined PEVs. Additionally, current PEVs research has utilized these vesicles therapeutically without extensively characterizing the resulting mitochondrial effects post-delivery. Therefore, additional research is necessary to determine the efficacy of PEVs in targeting and repairing dysfunctional mitochondria. Furthermore, exploring the potential of PEVs as drug carriers for mitochondria-focused therapies could open new avenues for treating mitochondrial-related diseases.

While some studies may support the presence of whole, intact mitochondria within PEVs, only a limited number have consistently observed them. Even when mitochondria are found, few detailed characterizations are conducted following mitochondrial internalization into PEVs. This is likely due to the diverse range of PEVs isolation and detection methods employed, which can make comparative analysis challenging. Although organizations, such as the International Society for Extracellular Vesicles (ISEV) and the EV-TRACK platform, have taken steps to standardize EVs isolation and detection, broader collaboration among research groups is essential to establish unified standards and regulations worldwide.
